# Neural Computations in a Dynamical System with Multiple Time Scales

**DOI:** 10.3389/fncom.2016.00096

**Published:** 2016-09-13

**Authors:** Yuanyuan Mi, Xiaohan Lin, Si Wu

**Affiliations:** ^1^Brain Science Center, Institute of Basic Medical SciencesBeijing, China; ^2^State Key Lab of Cognitive Neuroscience and Learning, IDG/McGovern Institute for Brain Research, Beijing Normal UniversityBeijing, China

**Keywords:** continuous attractor neural networks, short-term plasticity, spike frequency adaptation, persistent activity, adaptation, anticipative tracking

## Abstract

Neural systems display rich short-term dynamics at various levels, e.g., spike-frequency adaptation (SFA) at the single-neuron level, and short-term facilitation (STF) and depression (STD) at the synapse level. These dynamical features typically cover a broad range of time scales and exhibit large diversity in different brain regions. It remains unclear what is the computational benefit for the brain to have such variability in short-term dynamics. In this study, we propose that the brain can exploit such dynamical features to implement multiple seemingly contradictory computations in a single neural circuit. To demonstrate this idea, we use continuous attractor neural network (CANN) as a working model and include STF, SFA and STD with increasing time constants in its dynamics. Three computational tasks are considered, which are persistent activity, adaptation, and anticipative tracking. These tasks require conflicting neural mechanisms, and hence cannot be implemented by a single dynamical feature or any combination with similar time constants. However, with properly coordinated STF, SFA and STD, we show that the network is able to implement the three computational tasks concurrently. We hope this study will shed light on the understanding of how the brain orchestrates its rich dynamics at various levels to realize diverse cognitive functions.

## 1. Introduction

The brain performs computations by updating its internal states in response to external inputs. Neurons, synapses, and circuits are the fundamental units for implementing brain functions. At the synapse level, neurons interact with each other to enhance or depress their responses. At the single neuron level, a neuron integrates synaptic inputs and generates spikes if its membrane potential crosses a threshold. At the network level, the topology of neuronal connection pattern shapes the overall population activity. Taken together, the dynamics of individual neurons, the efficacy of synapses, and the network structure jointly determine the dynamical behavior of a neural system in response to external inputs which consequently determine/restrict the computations a neural system can perform. Thus, understanding the dynamical properties of neural systems and their roles in neural computations is at the core of using mathematical models to elucidate brain functions (Herz et al., 2006).

Experimental data has revealed that neural systems display rich dynamical behaviors. For instance, at the single neuron level, in addition to voltage-gated sodium and potassium currents for generating action potentials, a neuron also initiates slow calcium-activated potassium currents, and the latter suppress neuronal responses to a prolonged stimulation, a property called spike-frequency adaptation (SFA) (Gutkin and Zeldenrust, 2014). At the synapse level, rather than being a constant, the efficacy of a synapse exhibits temporal changes depending on the firing history of the pre-synaptic neuron, a property called short-term plasticity (STP) (Markram et al., 1998; Dittman et al., 2000; Abbott and Regehr, 2004). At the network level, experience-dependent long-term plasticity of synapses reshapes the connectivity of a network, creating new memory states in the system (Bliss and Collingridge, 1993). These rich dynamical features of neural networks form the basis for the brain to carry out various computational tasks.

Building up a neural network model and elucidating how the network dynamics reproduces experimental data is a common practice in computational neuroscience research. However, conventional modeling studies often focus on exploring how a single neural computation is realized by certain dynamical features of a neural circuit, albeit that in reality the same neural circuit is often engaged in many computational tasks. This is not a trivial issue, since the dynamical features needed for implementing different computations may be contradictory to each other, casting doubt on the feasibility of the model in practice. For instance, persistent activity and adaptation are two such computational tasks requiring seemingly conflicting neural dynamics. Persistent activity, referring to the phenomenon that neurons keep firing after removing the stimulation, is widely regarded as the neural substrate of short-term memory (Amit, 1989; note that here our definition of persist activity refers to the general phenomenon of sustained neuronal response after the stimulation is removed, and it is not limited to working memory). Adaptation, referring to the phenomenon that neuronal firing rates attenuate over time in response to an invariant stimulation, is generally believed to encompass a strategy for neural systems utilizing resources efficiently (Laughlin, 1989; Wark et al., 2007). To achieve persistent activity, it essentially requires a positive feedback loop in neuronal interaction which retains neural activity in the absence of external drive (Wang, 2001; Carter and Wang, 2007); whereas, to achieve adaptation, it requires a negative feedback loop which suppresses neural activity in the presence of external drive (Abbott et al., 1997). Thus, to achieve both persistent activity and adaptation in a single neural circuit, conflicting requirements on positive and negative feedbacks need to be properly reconciled.

In this work, we propose that the brain exploits different time scales of different dynamical features to accommodate contradictory computational requirements in a single neural circuit. To demonstrate this idea, we employ continuous attractor neural networks (CANNs) as the working model. CANNs are recurrent networks that can hold a family of stationary states due to their translation-invariant property, and they have been widely used as a canonic model to describe the encoding of continuous variables, such as orientation (Ben-Yishai et al., 1995), moving direction (Georgopoulos et al., 1993), head direction (Zhang, 1996), and spatial location of objects (Samsonovich and McNaughton, 1997), in neural systems. They have also been used as models to study working memory and navigation behaviors of animals (Taube, 2007). Recent experimental data has shown that CANNs capture some fundamental features of neural information representation (Mante et al., 2013; Ponce-Alvarez et al., 2013; Wimmer et al., 2014), suggesting that CANNs serve as a good mathematic model to investigate the general principles of neural computation (Wu et al., 2016). Based on CANNs, we investigate how coordination of SFA in the neuronal dynamics and STP in the synapse dynamics [which is further divided into short-term facilitation (STF) and short-term depression (STD)] enables a CANN to implement three different tasks, which are persistent activity, adaptation, and anticipative tracking.

In the literature, a large volume of theoretical studies has revealed that when short-term dynamics, such as STD, STF, or SFA, are included in a CANN, new interesting dynamical behaviors emerge, such as population spike, adaptive response, spontaneous traveling wave, and anticipative tracking, and that these new dynamical properties lead to new computational powers of a network (see e.g., Kilpatrick and Bressloff, 2009; York and Rossum, 2009; Ermentrout et al., 2010; Kilpatrick and Bressloff, 2010a,b; Itskov et al., 2011; Bressloff, 2012; Fung et al., 2012; Miller, 2013; Miller and Katz, 2013; Tsodyks and Wu, 2013; Fung and Amari, 2015 and references therein). The study of Renart et al. (2003) also showed that activity-dependent homeostatic scaling of synaptic strengths helps a CANN with heterogeneity in synapses to hold persistent activity reliably. The study of Barak and Tsodyks (2007) investigated systematically how different combinations of STD and STP result in qualitatively different traces of a network reaching to persistent activity, which suggests that the varied network responses mediated by STP can encode time-dependent stimuli. Compared to those previous studies, the contribution of the present study is mainly a demonstration that coordination of multiple short-term dynamics with different time scales can enable a single neural circuit to implement contradictory computational tasks.

## 2. Materials and methods

We employ two-dimensional CANNs as our working model to demonstrate the effects of different dynamical features. Consider that neurons are uniformly distributed in a two-dimensional space (*x, y*) with *x, y*∈(−∞, ∞). Denote *U*(**x**, *t*) to be the synaptic input to the neuron at the position **x** = (*x, y*), and *r*(**x**, *t*) the neuronal firing rate.The dynamics of the network is given by,

(1) $$\begin{array}{l} \tau \frac{\partial U(x,t)}{\partial t}=-U(x,t)+\rho \int_{-\infty }^{\infty }\int_{-\infty }^{\infty }J(x,x^{\prime })f(x^{\prime },t)p(x^{\prime },t)\\ \;r(x^{\prime },t)dx^{\prime }\\ \;-\;V(x,t)+g(x,t)I^{\text{ext}}(x,t), \end{array}$$

where τ denotes the synaptic time constant, which is typically in the order of 1−2 ms. ρ denotes the neuronal density. The variables *f*(**x**, *t*) and *p*(**x**, *t*) denote the STF and STD effects, respectively. *J*(**x**, **x′**) is the interaction strength between the neurons at **x** and **x′**, which is set to be

(2) $$J(x,x^{\prime })=\frac{J_{0}}{2\pi a^{2}}\text{exp}\left[-\frac{{\left(x-x^{\prime }\right)}^{2}+{\left(y-y^{\prime }\right)}^{2}}{2a^{2}}\right],$$

where the parameter *a* controls the range of neuronal interaction. Note that *J*(**x**, **x′**) denotes translation-invariant in the space, in terms of that it is a function of (**x − x′**), rather than **x**.

The firing rate of a neuron is determined by its received synaptic input according to

(3) $$r(x,t)=\frac{{\left[U(x,t)\right]}_{+}^{2}}{1+k\rho \int_{-\infty }^{\infty }\int_{-\infty }^{\infty }{\left[U(x^{\prime },t)\right]}_{+}^{2}dx^{\prime }},$$

where [.]_+_ is the rectifying function. The firing rate first increases with the synaptic input and then saturates gradually due to normalization by the total network activity. The latter can be realized by shunting inhibition, with the parameter *k* denoting inhibition strength (Hao et al., 2009; Zhang et al., 2013).

The network model exhibits a number of short-term dynamical features.

### Short-term facilitation in recurrent connection

The variable *f*(**x**, *t*) on the right-hand side of Equation (1) represents STF in recurrent interactions, whose dynamic is given by

(4) $$\tau_{f}\frac{\partial f(x,t)}{\partial t}=f_{min}-f(x,t)+\alpha \left[1-f(x,t)\right]r(x,t),$$

where τ_*f*_ is the time scale of STF and the parameter α controls the amplitude of the STF effect. The variable *f*(**x**, *t*) increases with neuronal firing rate, whose minimum value is *f*_*min*_ and maximum value is 1.

### Short-term depression in recurrent connection

The variable *p*(**x**, *t*) on the right-hand side of Equation (1) represents STD in recurrent interactions, whose dynamic is given by

(5) $$\tau_{d}\frac{\partial p(x,t)}{\partial t}=1-p(x,t)-\beta f(x,t)p(x,t)r(x,t),$$

where τ_*d*_ is the time scale of STD and the parameter β controls the amplitude of the STD effect. The variable *p*(**x**, *t*) decreases with neuronal firing rate, whose minimum value is 0 and maximum value is 1.

### Short-term depression in feedforward connection

The variable *g*(**x**, *t*) on the right-hand side of Equation (1) represents STD in feedforward connections to the neuron. Its product with the raw external input, *g*(**x**, *t*)*I*^ext^(**x**, *t*), gives the diminished feedforward synaptic current to the neuron. Its dynamic is given by

(6) $$\tau_{g}\frac{\partial g(x,t)}{\partial t}=1-g(x,t)-\eta g(x,t)I^{\text{ext}}(x,t),$$

where τ_*g*_ is the time scale of STD and the parameter η controls the amplitude of the STD effect. The variable *g*(**x**, *t*) decreases with external input *I*^ext^(**x**, *t*), whose minimum value is 0 and maximum value is 1.

### Spike frequency adaptation at single neuron

The current *V*(**x**, *t*) on the right-hand side of Equation (1) represents the effect of SFA, whose dynamic is given by

(7) $$\tau_{v}\frac{\partial V(x,t)}{\partial t}=-V(x,t)+m{\left[U\left(x,\;t\right)\right]}_{+},$$

where τ_*v*_ is the time scale of SFA and the parameter *m* controls the amplitude of the SFA effect. The solution of the above equation gives $V(x,t)=m\int_{-\infty }^{t}\text{exp}\left[-(t-t^{\prime })/\tau_{v}\right]U(x,t^{\prime })dt^{\prime }/\tau_{v}$, implying that *V*(**x**, *t*) is determined by the averaged neural activity over a period of τ_*v*_. The higher the neural activity, the larger is the negative feedback.

### The external input

The external input used in the present study is given by

(8) $$I^{\text{ext}}(x,t)=A_{\text{amp}}\text{exp}\left[-\frac{{\left(x-v_{\text{ext}}t\cos\theta \right)}^{2}+{\left(y-v_{\text{ext}}t\sin\theta \right)}^{2}}{4a^{2}}\right],$$

where *A*_*amp*_ is the input strength, *v*_*ext*_ the speed of moving input and θ the moving direction. For studying persistent activity, we apply a transient input to the network, which is obtained by setting *A*_*amp*_ > 0 for a short-time interval and *v*_*ext*_ = 0. For studying adaptation, we apply a sustained input to the network, which is obtained by setting *A*_*amp*_ to be a positive constant and *v*_*ext*_ = 0. For studying anticipative tracking, we apply a moving input to the network, which is obtained by setting *A*_*amp*_ to be a positive constant and *v*_*ext*_≠0.

## 3. Results

### 3.1. Network dynamics with individual dynamical features

To start, we first review the effect of each individual dynamical feature on the network dynamics. It can be checked that without these dynamical features (by setting α = β = *m* = η = 0), a CANN can hold a continuous family of Gaussian-shape stationary states (called bumps) in the absence of external drive (*I*^*ext*^ = 0), when the global inhibition strength *k* is below a critical value$k_{c}\equiv \rho {\left(J_{0}f_{min}\right)}^{2}/\left(32\pi a^{2}\right)$ (Fung et al., 2010). These stationary states are written as $U\left(x,t\right)=A_{u}\text{exp}\left[-{\left(x-q\right)}^{2}/\left(4a^{2}\right)\right]$, where the peak position of the bump **q** = (*q*_*x*_, *q*_*y*_) is a free parameter.

#### 3.1.1. The effect of STF

**Figure 1:**
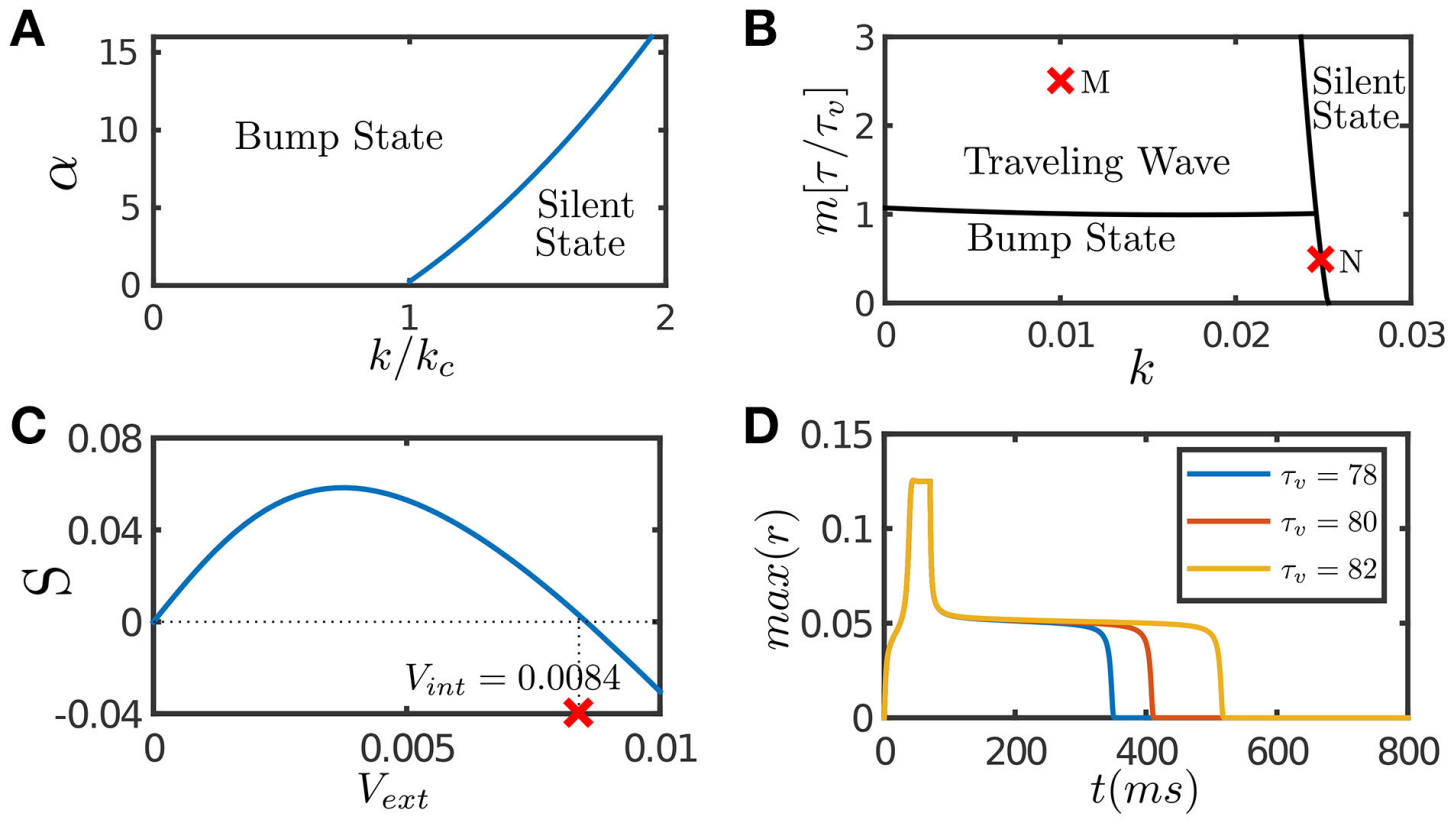
**Network dynamics with individual features**. **(A)** The phase diagram of a CANN with only STF included. The graph shows the stationary states of the network with the varying STF strength α and the global inhibition strength *k*. *k*_*c*_ is the critical inhibition strength below which a CANN without STF can hold bump states. STF allows the regime of bump states to expand to *k* > *k*_*c*_. **(B)** The phase diagram of a CANN with only SFA included. The network can hold traveling waves if the strength of SFA satisfies *m* > τ/τ_*v*_. **(C)** The tracking performance of a CANN with SFA. An external moving input given by Equation (8) is applied to the network, *A*_amp_ = 0.1. *S* is the separation between the network bump and the external input when tracking reaches to a stationary process. *Sv*_ext_ > 0 implies anticipative tracking. The parameters (*k, m*) = (0.01, 2.5) at the point M in **(B)**, where the network holds traveling waves, is used. *v*_int_ denotes the speed of the traveling wave the network can hold without relying on external drive. Anticipation occurs when *v*_ext_ < *v*_int_. **(D)** The plateau decay of a CANN with SFA when the network is marginally unstable. The parameters (*k, m*) = (0.0249, 0.5) at the point N close to the boundary in **(B)** is used. The lifetime of the plateau increases with the time scale τ_*v*_ of SFA. The simulations were done with a 2D CANN having 100 × 100 neurons uniformly distributed in the space (−π, π] with the periodic condition. Other parameters are *J*_0_ = 0.05, *a* = 0.5, τ = 1 ms.

In response to an external input, the effect of STF is to enlarge the interaction between neurons temporally, which enhances the positive feedback loop between neurons, and hence STF provides a mechanism to retain neural activity after removing the input. Figure 1A presents the phase diagram of a CANN with only STF included, which shows that STF enlarges the parameter regime for the network to hold active bump states.

#### 3.1.2. The effect of STD

The effect of STD is to depress the interaction between neurons temporally. For recurrent connections, STD weakens the positive feedback loop between neurons and hence has the effect of suppressing STF-triggered neural activity. For feedforward connections, STD reduces the synaptic input to a neuron and hence has the effect of suppressing the neuronal response to a prolonged stimulation. STD and SFA have similar effects on the CANN dynamics, except that the former operates at the synapse level and the latter at the neuron level. For simplicity, we only present the result for SFA (see below). The result for STD can be straightforwardly deduced.

#### 3.1.3. The effect of SFA

SFA induces an activity-dependent negative current to a neuron, which serves as a self-inhibition mechanism to suppress the neuronal response if a neuron has experienced prolonged firing. The phase diagram of a CANN with only SFA is presented in Figure 1B. Note that SFA induces a new form of stationary state, called traveling wave state (Ben-Yishai et al., 1997; York and Rossum, 2009; Bressloff, 2012; Tsodyks and Wu, 2013). In such a state, the network holds a spontaneously moving bump without relying on external drive. This spontaneous moving activity may be related to the traveling wave phenomenon widely observed in experiments (Wu et al., 2008). This dynamical property is also intuitively understandable. Suppose that a bump is initiated at a position. Due to SFA, those neurons which are most active are desensitized by the strongest negative feedback, and their activities will be suppressed consequently. With competition from neighboring neurons which are less affected by SFA, the bump tends to shift to the neighborhood; and at the new location, SFA starts to suppress neuronal responses again. Thus, the bump will keep propagating in the network like a traveling wave. Interestingly, when a CANN is within the parameter regime having a traveling wave solution, the network response to an external moving input will lead the input's instant position, if the speed of the moving input is smaller than that of the traveling wave the network can hold in the absence of external drive (Mi et al., 2014). This result is shown in Figure 1C, and the detailed mathematical analysis is presented in Appendix (Supplementary Material). Overall, the effect of SFA is to induce mobility to the network state, such that the network can track a moving input anticipatively. An illustration of this anticipative tracking behavior is presented in Figure 1A, and the computational role of anticipative tracking is discussed in Section 3.2.3.

In the parameter regime close to the boundary between active and silent states (e.g., the point N in Figure 1B), the network dynamics displays another interesting phenomenon, called the plateau decay behavior (see Figure 1D), that is, starting from an initial active state, the network activity will decay very slowly in the time scale of SFA, followed by a rapid fall. The detailed analysis of this plateau decay behavior is presented in the Appendix (Supplementary Material), and its computational role is discussed in Section 4.1. Apparently, since the effect of STD is to induce negative feedback modulation similar to that of SFA, STD can also generate the plateau decay behavior if its amplitude is properly chosen (Fung et al., 2012).

### 3.2. Network dynamics with combined short-term dynamical features

We now come to study how combined dynamical features with different time scales endow a CANN with the capacity of implementing three contradictory computational tasks, which are: (1) retaining persistent neural firing for a considerable amount of time after removing the stimulation, (2) generating adaptive neural responses to a prolonged invariant stimulation, and (3) tracking an external moving input anticipatively.

As described above, the effects of STF and STD or SFA on the network dynamics are opposite to each other. If we simply combine them together with the same time scale, a CANN is unable to implement three computational tasks concurrently. However, by assigning different time scales for them, a CANN may be able to achieve this goal. For instance, by setting the time constants τ_*f*_ ≪ τ_*d*_ (note other parameters, such as the neuronal connection strengths *J*(**x, x′**), also need to be set properly, see e.g., the analysis in Barak and Tsodyks, 2007), a network can, on one hand, enhance the interaction between neurons in a shorter time scale τ_*f*_ necessary for generating persistent activity, and, on the other hand, reduce the interaction in a larger time scale τ_*d*_ necessary for adaptation and closing-down neural activity. The effects of STD and SFA on the network dynamics are similar, which essentially produce negative feedback modulation, but by including both of them with different time scales, a CANN is able to exploit multiple computational properties associated with the negative feedback modulation.

Below we show that if the time scales of different dynamical features satisfy the condition,

(9) $$\tau \ll \tau_{f}\ll \tau_{v}\ll \tau_{d}\ll \tau_{g},$$

the CANN is able to implement the above three contradictory computational tasks. It is known that there exist large diversities for STP and SFA in different cortical regions and for different neuron types. The parameters we consider here tend to hold in the sensory cortex where the synapses are STD-dominating (Wang et al., 2006; Gutkin and Zeldenrust, 2014).

#### 3.2.1. Persistent activity

Persistent activity refers to the fact that neurons keep firing after removing the external drive, a property widely regarded as the neural substrate of short-term memory (Amit, 1989). With the condition Equation (9), our network model can reproduce this phenomenon.

**Figure 2:**
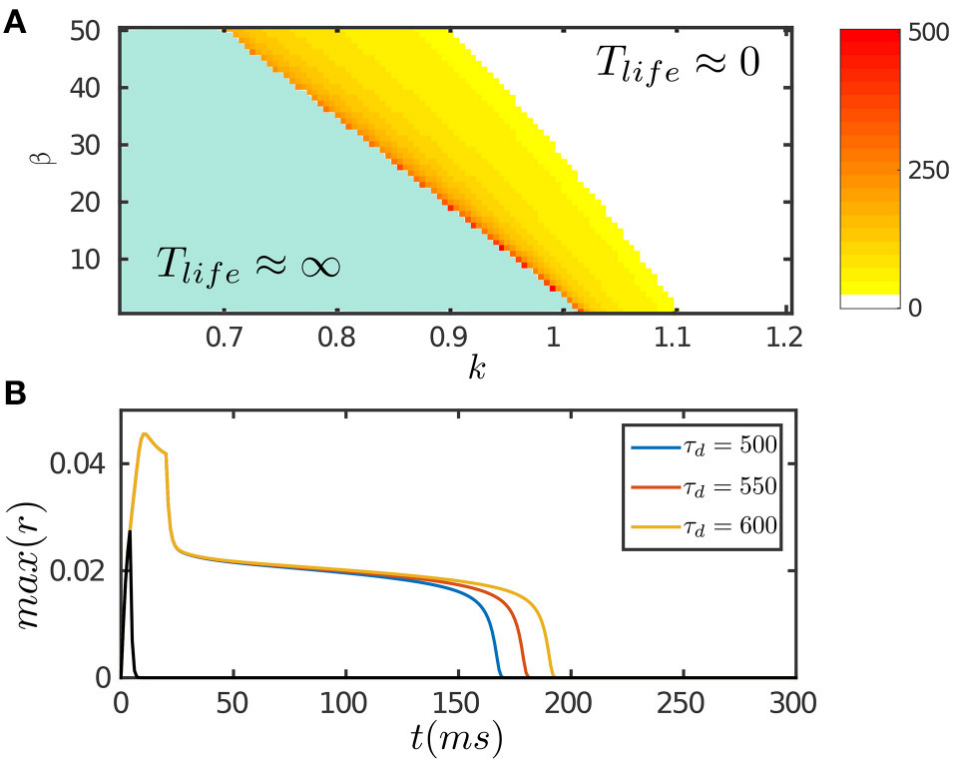
**Persistent activity of the network in response to a transient input**. The input is given by Equation (8). **(A)** The phase diagram of the network with varying STD strength β and inhibition strength *k*. The network can hold persistent activity of finite lifetimes over a range of parameter values around the boundary. The lifetime of persistent activity, *T*_*life*_, is measured by the length of the decaying plateau. **(B)** The lifetime of persistent activity with the varying time scale of STD τ_*d*_. β = 100. *k* = 0.12. The network holds persistent activity if the stimulation duration is long enough in the order of τ_*f*_; otherwise the network response fades away rapidly as indicated by the black curve. Other parameters are: *a* = 0.5, *J*_0_ = 0.5, *f*_*min*_ = 0.1, α = 20, *m* = 2.5τ/τ_*v*_, η = 40, τ = 1 ms, τ_*f*_ = 10 ms, τ_*v*_ = 80 ms, τ_*g*_ = 3500 ms, *A*_amp_ = 0.15.

The results are presented in Figure 2. Figure 2A displays the phase diagram of the network with varying STD and inhibition strengths, which shows that the network holds persistent activity of finite lifetimes over a range of parameters around the boundary where the network is marginally unstable. In response to a transient input, the network first generates a strong response due to facilitation of the interaction between neurons via STF. If the stimulation duration is long enough, up to the order of τ_*f*_, the neuronal recurrent connections will be sufficiently facilitated to retain neural activity without relying on external drive; otherwise, the neural activity fades away rapidly (Figure 2B). As time goes on, the negative feedback modulation via both SFA and STD begins to dominate and eventually suppresses the neural activity. We require that the amplitudes of SFA and STD satisfy two requirements: (1) the amplitude of SFA alone, which dominates on the time scale of τ_*v*_ (τ_*f*_ ≪ τ_*v*_ ≪ τ_*d*_), is not adequate to suppress the neural activity, so that persistent activity lasts over the time scale τ_*v*_. This condition is also required for implementing anticipative tracking introduced below. (2) The amplitude of STD, which dominates on the time scale of τ_*d*_ (τ_*d*_ ≫ τ_*v*_), is in the parameter regime where the network is marginally unstable (Figure 2A), so that the network activity decays very slowly like a plateau on the time scale of τ_*d*_ and then falls rapidly (Figure 2B). We interpret this plateau decay as persistent activity of a finite lifetime. The plateau decay behavior is analyzed for SFA as shown in Figure 1D and with details given in Appendix (Supplementary Material). Since SFA and STD have similar effects on the network dynamics, it is understandable that STD also holds this property.

Note that we here consider a mechanism using STD to realize short-term memory in a network, and the duration of this kind of short-term memory is in the time scale of STD (Figure 2B). This mechanism has the advantage that it does not require an extra operation to turn-off the network activity (Gutkin et al., 2001). Apparently, there exist other types of short-term memory with different time scales in the brain, such as working memory in the prefrontal cortex whose duration lasts from seconds to minutes. These different types of short-term memory may recruit different mechanisms to hold and turn-off neuronal persistent activities.

#### 3.2.2. Adaptation

Adaptation refers to the phenomenon that neurons dynamically adjust their response properties in accordance with the statistics of the external inputs. It has been widely suggested that adaptation encompasses a strategy for a neural system to utilize its resources (such as spikes) efficiently to encode input information (Laughlin, 1989; Wark et al., 2007). In luminance adaptation, in which the visual system adapts to a sustained stimulation of constant luminance, neuronal responses exhibit a stereo-typed temporal characteristic: at the onset of the stimulation, neuronal responses increase dramatically; and afterwards, neuronal responses attenuate gradually down to a level close to background activity (Boynton and Whitten, 1970). With the condition Equation (9), our model can reproduce this phenomenon.

**Figure 3:**
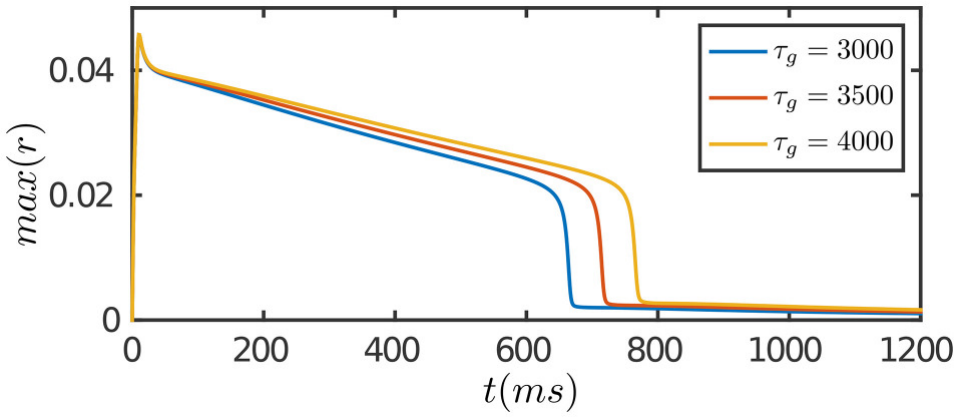
**Adaptation of the network in response to a constant input**. The input is given by Equation (8). The duration of adaptation, measured from the onset of stimulation to the moment the network activity fades away, is determined by the time scale of STD in feedforward connection, τ_*g*_. Other parameters are the same as Figure 2.

The results are presented in Figure 3. At the onset of the stimulation, the network first generates a strong transient response due to facilitation of neuronal interaction via STF. As time goes on, SFA and STD in recurrent connections begin to dominate, which suppress neuronal responses. Furthermore, STD in feedforward connections takes effect on the time scale of τ_*g*_, which depresses the feedforward inputs to neurons. The overall effect is that neuronal responses attenuate over time, although the external input remains invariant, and eventually the network activity reaches to a level close to background activity. Compared to the case of persistent activity, STD in feedforward connections plays a crucial role in adaptation which diminishes the feedforward current to a neuron for large external inputs.

#### 3.2.3. Anticipative tracking

Anticipative tracking refers to the phenomenon that a neural system smoothly tracks the changing of a moving stimulus in a manner that the neural representation leads the actual position of the stimulus. This phenomenon has been widely observed in retinal neurons (Leonardo and Meister, 2013), and head direction (HD) neurons when animals are navigating in space (Taube, 2007). For instance, in anterior dorsal thalamic nuclei of rodent, it was found that the head-direction encoded by HD neurons led the actual instant direction of the rat head by around 20 ms, i.e., the neural representation pointed to the angle the rat head would turn into 20 ms later (Blair et al., 1997). Similarly, anticipation occurs when our eyes smoothly pursuit moving objects (Heinen and Liu, 1997). Anticipative tracking is fundamental for the brain to process motion information, since transmission delays of neural signals are significant and need to be compensated, e.g., the delay for neural signal transmitted from retina to V1 takes about 50~80 ms (Nowak et al., 1995). With the condition Equation (9), our model can reproduce this phenomenon.

**Figure 4:**
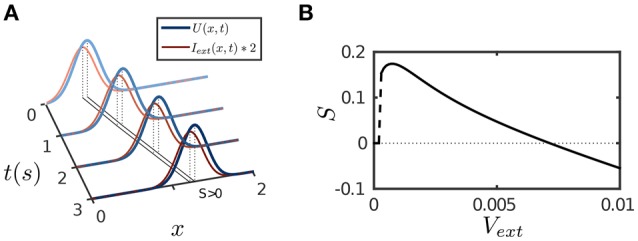
**Tracking performance of the network**. The moving input is given by Equation (8). **(A)** An example of anticipative tracking of the network. *v*_ext_ = 0.8/*s*. For the illustration purpose, the 2D bump is projected on the normal of the moving direction, and the external input strength is scaled up by twofold. **(B)** The condition for anticipative tracking. Anticipation occurs when *Sv*_ext_ > 0, where *S* is the separation between the network bump and the external input. When the speed is too small, *S* = 0 implies no tracking at all; and when the speed is too large, *Sv*_ext_ < 0 implies lagging. Other parameters are the same as Figure 2.

The results are presented in Figure 4. The parameter values are the same as for implementing persistent activity and adaptation. For a transient or a static input, since STD has the longest time scale τ_*d*_, its effect will eventually dominate the network dynamics and suppress neural activity as described above. However, for a moving input, once the network bump moves away on the time scale of τ_*v*_ (τ_*v*_ ≪ τ_*d*_) to track the input, the effect of depressed synapses at the old location can be neglected, thus, the network dynamics can be largely understood as if the CANN only includes SFA; and the latter is analyzed in Figures 1B,C. We therefore set the amplitude of SFA in the parameter range where the network holds traveling waves and is able to achieve anticipative tracking. Figure 4A displays an example of anticipative tracking of the network. Figure 4B presents the condition for anticipative tracking. Note that when the speed of the moving input is too small, no tracking occurs, since STD suppresses the neural activity before it starts to move.

## 4. Discussion

Neural systems display rich short-term dynamics at all levels, from neurons to synapses and to circuits. These dynamical features cover a broad range of time scales and exhibit large diversity in different cortical regions. For instances, the time constants of SFA for different neuron types and in different areas expand from tens to thousands of milliseconds (Benda and Herz, 2003); the time constants of STP cover a similar range but the relative sizes between STF and STD vary in different cortical areas, e.g., STF is dominating and has a longer time constant in the prefrontal cortex; whereas, STD is dominating and has a longer time constant in the sensory cortex (Wang et al., 2006). It remains largely unclear what the purpose for the brain is to hold such variabilities in dynamical features and their time scales.

In the present study, we argue that one benefit for having multiple dynamical features with varied time scales is that the brain can fully exploit the advantages of these features to implement what are otherwise contradictory computational tasks. To demonstrate this idea, we consider STF, SFA, and STD with increasing time constants in the dynamics of a CANN, and show that the network is able to implement three seemingly contradictory computations, which are persistent activity, adaptation and anticipative tracking. Simply stated, the role of STF is to hold persistent activity in the absence of external drive, the role of SFA is to support anticipative tracking for a moving input, and the role of STD is to eventually suppress neural activity for a static or transient input. Notably, the time constants of SFA and STD can be swapped with each other, since SFA and STD have similar effects on the network dynamics. Nevertheless, we need to include both of them, or only one of them (either SFA or STD) but with multiple time scales, since a single negative feedback modulation with a constant time scale is unable to achieve both anticipative tracking and plateau decay concurrently. The implementation of each individual computational task based on a single or combined dynamical features has been studied previously (e.g., Barak and Tsodyks, 2007; Fung et al., 2012). Here, our main contribution is a demonstration that contradictory computational tasks can be realized concurrently in a single neural circuit if different short-term dynamics are combined properly.

In the present study, we have considered STP and SFA with multiple time scales. Alternatively, a neural system may have other mechanisms to realize short-term dynamics of varied time scales. For instance, the work of (Goldman, 2009) considered heterogeneities in synapse strengthes and neuronal connections among neuronal groups, such that different groups have different time constants in response to a transient external input, and a read-out neuron can integrate the responses of different neuronal groups over time to achieve persistent firing. It will be interesting to explore whether such a mechanism can also implement the three computational tasks considered in this study concurrently.

Finally, we should point out that we have not found direct experimental evidence confirming that a single cortical region realizes three computational tasks considered in this study. However, based on the known experimental data, we expect that this is very likely to be true. For instance, the primary visual cortex V1 may realize these three computations. It is known that in the sensory cortex, neuronal synapses are STD-dominating, fitting the parameter condition Equation (9), and that neurons in V1 exhibit adaptive behaviors, have the capacity to track a moving stimulus anticipatively (Xu et al., 2012), and can hold long-lasting residual activities in response to a transient stimulation (Jancke et al., 2004). Further experimental studies are needed to validate our hypothesis. Nevertheless, we hope that this study can shed light on our understanding of how the brain orchestrates its rich dynamics at various levels to realize diverse cognitive functions.

## Author contributions

YM and SW conceived the project idea. XL implemented the simulation. YM and SW performed the theoretical analysis. XL, YM, and SW wrote the paper.

### Conflict of interest statement

The authors declare that the research was conducted in the absence of any commercial or financial relationships that could be construed as a potential conflict of interest.
