# Supplementary material for: Neural Computations in a Dynamical System with Multiple Time Scales
Source: Front Comput Neurosci. 2016 Sep 13;10:96. doi: 10.3389/fncom.2016.00096 (PMC5020071; doi:10.3389/fncom.2016.00096)
Supplement: Supplementary file 1 [file Appendix.pdf]

Appendix:
Neural Computations in a Dynamical System with Multiple Time Scales

Yuanyuan Mi, Xiaohan Lin and Si Wu
*Correspondence:
Author Name: Si Wu
wusi@bnu.edu.cn

DYNAMICS OF A TWO-DIMENSIONAL CANN WITH SFA

The dynamics of a 1D CANN with SFA was analyzed previously (Mi et al., 2014). Here, we solve the dynamics of a 2D CANN with SFA.

When only SFA is included, the dynamics of a 2D CANN is written as (compared to Eqs.(1, 3, 7) in the main text),

$$\tau \frac{\partial U(\mathbf{x},t)}{\partial t} = -U(\mathbf{x},t) + \rho \int_{-\infty}^{+\infty}\int_{-\infty}^{+\infty} J(\mathbf{x};\mathbf{x}')r(\mathbf{x}',t)d\mathbf{x}' - V(\mathbf{x},t) + I^{\text{ext}}(\mathbf{x},t), \tag{S1}$$

$$\tau_v \frac{\partial V(\mathbf{x},t)}{\partial t} = -V(\mathbf{x},t) + m[U(\mathbf{x},t)]_+, \tag{S2}$$

$$r(\mathbf{x},t) = \frac{[U(\mathbf{x},t)]_+^2}{1 + k\rho \int_{-\infty}^{+\infty}\int_{-\infty}^{+\infty} [U(\mathbf{x}',t)]_+^2 d\mathbf{x}'}. \tag{S3}$$

By numerically solving the equations above, we can obtain the phase diagram of the network, as shown in Fig.1B.

Plateau decay of network activity

In the parameter regime where the network can only hold the silent state but is close to the boundary separating the active states (e.g., the point N in Fig.1B), the network dynamics displays an interesting phenomenon, that is, the network activity decays very slowly on the time scale of SFA. Below we analyze this plateau decay behavior.

We consider that during the decay the network state still has the Gaussian-shape (a good approximation confirmed by simulation), but the height of the bump changes over time, which is given by

$$\overline{U}(\mathbf{x},t) = A_u(t)\text{exp}\left[-\frac{(x-q_x)^2+(y-q_y)^2}{4a^2}\right], \tag{S4}$$

$$\overline{r}(\mathbf{x},t) = A_r(t)\text{exp}\left[-\frac{(x-q_x)^2+(y-q_y)^2}{2a^2}\right], \tag{S5}$$

$$\overline{V}(\mathbf{x},t) = A_v(t)\text{exp}\left[-\frac{(x-q_x)^2+(y-q_y)^2}{4a^2}\right], \tag{S6}$$

where $\mathbf{q} = (q_x, q_y)$ denotes the bump position.

Substituting Eqs. (S4-S6) into Eq. (S1-S2), we get

$$\tau \frac{dA_u}{dt} = -A_u + \frac{\rho J_0 A_r}{2} - A_v, \tag{S7}$$

$$\tau_v \frac{dA_v}{dt} = -A_v + mA_u, \tag{S8}$$

$$A_r = \frac{A_u^2}{1 + 2\pi k \rho a^2 A_u^2}. \tag{S9}$$

For the illustrative purpose, we only study the dynamics of bump height A_u and assume that A_v reaches to its steady value instantly. By setting $dA_v/dt = 0$ in Eq.(S8) and substituting it into Eq.(S7), we obtain,

$$\tau \frac{dA_u}{dt} = -(1+m)A_u + \frac{\rho J_0 A_u^2}{2 + 4\pi k \rho a^2 A_u^2} \equiv F(A_u). \tag{S10}$$

At the boundary between the silent and active states, the network holds a stable state $A_u = 0$ and an unstable state $A_u^* = 4(1+m)/(\rho J_0)$ (obtained by solving $F(A_u) = 0$). Close to the boundary, the function $F(A_u)$ has the form as illustrated in Fig.S1. We see that starting from a state $A_u^0 > A_u*$, the decay of the bump height will take a considerable amount of time to cross the point A_u^*, since in this region, the decaying speed of the bump height given by $F(A_u)$ is close to zero (see Fig.1D).

The time T consumed for the network crossing the point A_u^* is estimated to be

$$\begin{aligned}
\int_0^T dt &= \int_{A_u^{*+}}^{A_u^{*-}} \frac{\tau}{F(A_u)} dA_u, \\
&\approx \int_{A_u^{*+}}^{A_u^{*-}} \frac{\tau}{F(A_u^*) + \frac{1}{2}(A_u - A_u*)^2 F''(A_u*)} dA_u, \\
&= \frac{2\tau}{\sqrt{2F(A_u^*)F''(A_u^*)}} \left[\tan^{-1} \frac{A_u^{*-}}{\sqrt{2F(A_u^*)/F''(A_u^*)}} - \tan^{-1} \frac{A_u^{*+}}{\sqrt{2F(A_u^*)/F''(A_u^*)}} \right], \\
&= \frac{2\tau}{\sqrt{2F(A_u^*)F''(A_u^*)}} G(A_u^*).
\end{aligned} \tag{S11}$$

where A_u^{*+} and A_u^{*-} denote, respectively, the points slightly larger or smaller than A_u^*, $F'(A_u^*) = dA_u/dt|_{A_u^*}$, and $F''(A_u^*) = dF'(A_u)/dt|_{A_u^*}$. To get the above results, we use the second-order Taylor expansion of $F(A_u)$ at A_u^*, and the condition $F'(A_u^*) = 0$.

In the limit of $F(A_u^*) \to 0$, the value of $G(A_u^*)$ is bounded. Thus, the time of the bump height decay is on the time scale of

$$T \propto \frac{2\tau}{\sqrt{2F(A_u^*)F''(A_u^*)}}. \tag{S12}$$

Traveling wave state

When SFA is strong, the negative feedback it generates will suppress localized neuronal responses, leading to an interesting phenomenon in the dynamics of a CANN. That is, the network can support a spontaneously moving bump without relying on an external drive. This phenomenon is called traveling wave. When the network bump is moving, its Gaussian shape is distorted. The higher the moving speed, the more severe the distortion is. For simplicity of analysis, we assume that the network state still has the Gaussian shape, which is assumed to be

$$\overline{U}(\mathbf{x},t) = A_u \mathrm{exp}\left[-\frac{(x-vt\cos\theta)^2+(y-vt\sin\theta)^2}{4a^2}\right], \tag{S13}$$

$$\overline{r}(\mathbf{x},t) = A_r \mathrm{exp}\left[-\frac{(x-vt\cos\theta)^2+(y-vt\sin\theta)^2}{2a^2}\right], \tag{S14}$$

$$\overline{V}(\mathbf{x},t) = A_v \mathrm{exp}\left[-\frac{(x-vt\cos\theta+d\cos\theta)^2}{4a^2}\right]\mathrm{exp}\left[-\frac{(y-vt\sin\theta+d\sin\theta)^2}{4a^2}\right], \tag{S15}$$

where v is the moving speed of the bump and $\theta = \arctan(y/x)$ is the moving direction. The parameter d denotes the separation between the peaks of the bumps $U(\mathbf{x},t)$ and $V(\mathbf{x},t)$ along the moving direction θ. The condition $vd > 0$ always holds, due to that the feedback of SFA is delayed.

To solve the traveling wave state of the network, we utilize an important property of CANNs, that is, the dynamics of a CANN is dominated by a few motion modes corresponding to different distortion features of the bump state. We can project the network dynamics on these dominating modes and simplify the network dynamics significantly (Fung et al., 2010). The first two dominating modes we use correspond to the distortions in the height and position of a bump. For the dynamics of $U(\mathbf{x},t)$ and $V(\mathbf{x},t)$, they are given by

$$\begin{aligned}
u_0(\mathbf{x}|\mathbf{q}) &= e^{-\left[(x-q_x)^2+(y-q_y)^2\right]/(4a^2)},\\
u_1(\mathbf{x}|\mathbf{q}) &= \cos\theta(x-q_x)e^{-\left[(x-q_x)^2+(y-q_y)^2\right]/(4a^2)}+\sin\theta(y-q_y)e^{-\left[(x-q_x)^2+(y-q_y)^2\right]/(4a^2)},\\
v_0(\mathbf{x}|\mathbf{q}-\mathbf{d}) &= e^{-\left[(x-q_x+d_x)^2+(y-q_y+d_y)^2\right]/(4a^2)},\\
v_1(\mathbf{x}|\mathbf{q}-\mathbf{d}) &= \cos\theta(x-q_x+d_y)e^{-\left[(x-q_x+d_x)^2+(y-q_y+d_y)^2\right]/(4a^2)}\\
&\quad+\sin\theta(y-q_y+d_y)e^{-\left[(x-q_x+d_x)^2+(y-q_y+d_y)^2\right]/(4a^2)},
\end{aligned}$$

where $q_x = vt\cos\theta$, $q_y = vt\sin\theta$, $d_x = d\cos\theta$ and $d_y = d\sin\theta$.

Substituting Eqs.(S13-S15) into Eq.(S1), we obtain

$$
\begin{aligned}
\text{Left-side} &= \tau A_u v \cos\theta \frac{x - vt\cos\theta}{2a^2} e^{-\left[(x-vt\cos\theta)^2+(y-vt\sin\theta)^2\right]/(4a^2)} \\
&+ \tau A_u v \sin\theta \frac{y - vt\sin\theta}{2a^2} e^{-\left[(x-vt\cos\theta)^2+(y-vt\sin\theta)^2\right]/(4a^2)}, \\
\text{Right-side} &= [-A_u + \frac{\rho J_0 A_r}{2}] e^{-\left[(x-vt\cos\theta)^2+(y-vt\sin\theta)^2\right]/(4a^2)} \\
&- A_v e^{-\left[(x-vt\cos\theta+d\cos\theta)^2\right]/(4a^2)} e^{-\left[(y-vt\sin\theta+d\sin\theta)^2\right]/(4a^2)}.
\end{aligned}
$$

Projecting both sides onto the motion mode $u_0(\mathbf{x}|\mathbf{q})$, we obtain

$$
-A_u + \frac{\rho J_0 A_r}{2} - A_v e^{-\frac{d^2}{8a^2}} = 0. \tag{S16}
$$

Projecting both sides onto the motion mode $u_1(\mathbf{x}|\mathbf{q})$, we obtain

$$
\tau A_u v = A_v d e^{-\frac{d^2}{8a^2}}. \tag{S17}
$$

Substituting Eqs.(S13-S15) into Eq.(S2), we obtain

$$
\begin{aligned}
\text{Left-side} &= \tau_v A_v v [\frac{x - vt\cos\theta + d\cos\theta}{2a^2}\cos\theta + \frac{y - vt\sin\theta + d\sin\theta}{2a^2}\sin\theta] \\
&\times e^{-\left[(x-vt\cos\theta+d\cos\theta)^2+(y-vt\sin\theta+d\sin\theta)^2\right]/(4a^2)}, \\
\text{Right-side} &= -A_v e^{-(x-vt\cos\theta+d\cos\theta)^2/(4a^2)} e^{-(y-vt\sin\theta+d\sin\theta)^2/(4a^2)} \\
&+ m A_u e^{-\left[(x-vt\cos\theta)^2+(y-vt\sin\theta)^2\right]/(4a^2)}.
\end{aligned}
$$

Projecting both sides onto the motion mode $v_0(\mathbf{x}|\mathbf{q}-\mathbf{d})$, we obtain

$$
A_v = m A_u e^{-\frac{d^2}{8a^2}}. \tag{S18}
$$

Projecting both sides onto the motion mode $v_1(\mathbf{x}|\mathbf{q}-\mathbf{d})$, we obtain

$$
\tau_v A_v v = m A_u d e^{-\frac{d^2}{8a^2}}. \tag{S19}
$$

Substituting Eqs. (S13,S14) into Eq. (S3), we get

$$
A_r = \frac{A_u^2}{1 + 2k\rho\pi a^2 A_u^2}. \tag{S20}
$$

Combining Eqs.(S16-S20), we get the bump heights, the separation distance d, and the speed of the traveling wave, which are

$$
\begin{aligned}
A_u &= \frac{\rho J_0 + \sqrt{\rho^2 J_0^2 - 32k\rho\pi a^2(1+\tau/\tau_v)^2}}{8k\rho\pi a^2(1+\tau/\tau_v)}, && \text{(S21)}\\
A_r &= \frac{\rho J_0 + \sqrt{\rho^2 J_0^2 - 32k\rho\pi a^2(1+\tau/\tau_v)^2}}{4k\rho^2 J_0 \pi a^2}, && \text{(S22)}\\
A_v &= \sqrt{\frac{m\tau}{\tau_v}}\frac{\rho J_0 + \sqrt{\rho^2 J_0^2 - 32k\rho\pi a^2(1+\tau/\tau_v)^2}}{8k\rho\pi a^2(1+\tau/\tau_v)}, && \text{(S23)}\\
v &\equiv v_{\text{int}} = \frac{2a}{\tau_v}\sqrt{ln\frac{m\tau_v}{\tau}}, && \text{(S24)}\\
d &= 2a\sqrt{ln\frac{m\tau_v}{\tau}}. && \text{(S25)}
\end{aligned}
$$

We see that the speed of the traveling wave (Eq.(S24)) is fully determined by network parameters, therefore we call it the intrinsic speed of the network, denoted as v_{int}.

The condition for the network to hold a traveling wave is given by

$$
m > \tau/\tau_v, \qquad 0 < k < \frac{\rho J_0^2}{32\pi a^2(1+\tau/\tau_v)^2}. \tag{S26}
$$

Tracking performance

We analyze the tracking performance of a 2D CANN with SFA in response to an external moving input. Without loss of generality, we consider that the external input has the form,

$$
I^{\text{ext}}(\mathbf{x}, t) = A_{amp}\exp\left[-\frac{(x - v_{\text{ext}}t\cos\theta)^2 + (y - v_{\text{ext}}t\sin\theta)^2}{4a^2}\right], \tag{S27}
$$

where A_{amp} denotes the input strength. The input moves at a constant speed v_{ext} in the direction θ.

Denote $\mathbf{S} = (S_x, S_y)$ the separation between the network bump and the external input. During the tracking, the network state still keeps the Gaussian-shape, which can be written as

$$
\begin{aligned}
\overline{U}(\mathbf{x}+\mathbf{S}, t) &= A_u\exp\left[-\frac{(x - S_x - v_{\text{ext}}t\cos\theta)^2 + (y - S_y - v_{\text{ext}}t\sin\theta)^2}{4a^2}\right], && \text{(S28)}\\
\overline{r}(\mathbf{x}+\mathbf{S}, t) &= A_r\exp\left[-\frac{(x - S_x - v_{\text{ext}}t\cos\theta)^2 + (y - S_y - v_{\text{ext}}t\sin\theta)^2}{2a^2}\right], && \text{(S29)}\\
\overline{V}(\mathbf{x}+\mathbf{S}, t) &= A_v\exp\left[-\frac{(x - S_x - v_{\text{ext}}t\cos\theta + d\cos\theta)^2}{4a^2}\right]\\
&\quad \times\exp\left[-\frac{(y - S_y - v_{\text{ext}}t\sin\theta + d\sin\theta)^2}{4a^2}\right]. && \text{(S30)}
\end{aligned}
$$

We consider that in the stationary state, the network is able to track the moving input, which means that the bump moves at the speed v_{ext} and the separation can be expressed as $\mathbf{S} = (S\cos\theta, S\sin\theta)$ with S a constant. The condition $v_{\text{ext}}S > 0$ implies that the bump is leading the external input; and otherwise is lagging behind.

We apply the projection method as described above to solve the tracking behaviors of the network. Substituting Eqs.(S28-S30) into Eq.(S1), we obtain

$$\begin{aligned}
\text{Left-side} &= \tau A_u v_{\text{ext}}\cos\theta \frac{x+S_x-v_{\text{ext}}t\cos\theta}{2a^2} e^{-\left[(x+S_x-v_{\text{ext}}t\cos\theta)^2+(y+S_y-v_{\text{ext}}t\sin\theta)^2\right]/(4a^2)} \\
&+\tau A_u v_{\text{ext}}\sin\theta \frac{y+S_y-v_{\text{ext}}t\sin\theta}{2a^2} e^{-\left[(x+S_x-v_{\text{ext}}t\cos\theta)^2+(y+S_y-v_{\text{ext}}t\sin\theta)^2\right]/(4a^2)}, \\
\text{Right-side} &= [-A_u + \frac{\rho J_0 A_r}{2}] e^{-\left[(x+S_x-v_{\text{ext}}t\cos\theta)^2+(y+S_y-v_{\text{ext}}t\sin\theta)^2\right]/(4a^2)} \\
&-A_v e^{-\left[(x+S_x-v_{\text{ext}}t\cos\theta+d\cos\theta)^2+(y+S_y-v_{\text{ext}}t\sin\theta+d\sin\theta)^2\right]/(4a^2)} \\
&+A_{amp} e^{-\left[(x-v_{\text{ext}}t\cos\theta)^2+(y-v_{\text{ext}}t\sin\theta)^2\right]/(4a^2)}.
\end{aligned}$$

Projecting both sides onto the motion mode $u_0(\mathbf{x}|\mathbf{q}-\mathbf{S})$, we obtain

$$-A_u + \frac{\rho J_0 A_r}{2} - A_v e^{-\frac{d^2}{8a^2}} + A_{amp} e^{-\frac{S^2}{8a^2}} = 0. \tag{S31}$$

Projecting both sides onto the motion mode $u_1(\mathbf{x}|\mathbf{q}-\mathbf{S})$, we obtain

$$\tau A_u v = A_v d e^{-\frac{d^2}{8a^2}} - A_{amp} S e^{-\frac{S^2}{8a^2}}. \tag{S32}$$

Substituting Eqs.(S28-S30) into Eq.(S2), we obtain

$$\begin{aligned}
\text{Left-side} &= \tau_v A_v v_{\text{ext}} [\frac{x+S_x-v_{\text{ext}}t\cos\theta+d\cos\theta}{2a^2}\cos\theta + \frac{y+s_y-v_{\text{ext}}t\sin\theta+d\sin\theta}{2a^2}\sin\theta] \\
&\times e^{-\left[(x+S_x-v_{\text{ext}}t\cos\theta+d\cos\theta)^2+(y+S_y-v_{\text{ext}}t\sin\theta+d\sin\theta)\right]/(4a^2)}, \\
\text{Right-side} &= -A_v e^{-\left[(x+S_x-v_{\text{ext}}t\cos\theta+d\cos\theta)^2+(y+S_y-v_{\text{ext}}t\sin\theta+d\sin\theta)^2\right]/(4a^2)} \\
&+m A_u e^{-\left[(x+S_x-v_{\text{ext}}t\cos\theta)^2+(y+S_y-v_{\text{ext}}t\sin\theta)^2\right]/(4a^2)}.
\end{aligned}$$

Projecting both sides onto the motion mode $v_0(\mathbf{x}|\mathbf{q}-\mathbf{S}-\mathbf{d})$ and equating both sides, we have

$$A_v = m A_u e^{-\frac{d^2}{8a^2}}. \tag{S33}$$

Projecting both sides onto the motion mode $v_1(\mathbf{x}|\mathbf{q}-\mathbf{S}-\mathbf{d})$ and equating both sides, we have

$$\tau_v A_v v = m A_u d e^{-\frac{d^2}{8a^2}}. \tag{S34}$$

Substituting Eqs. (S28,S29) into Eq. (S3), we get

$$A_r = \frac{A_u^2}{1 + 2k\rho\pi a^2 A_u^2}. \tag{S35}$$

Combining Eqs.(S31 - S35), the network dynamics is solved, which gives

$$d = \tau_v v_{\text{ext}}, \tag{S36}$$

$$Se^{-\frac{S^2}{8a^2}} = \frac{A_u v_{\text{ext}} m \tau_v}{A_{amp}} \left[e^{-\frac{\tau_v^2 v_{\text{ext}}^2}{4a^2}} - \frac{\tau}{m\tau_v} \right], \tag{S37}$$

From Eqs. (S37,S24), we see that the condition for $S v_{\text{ext}} > 0$, i.e., the network state leads the input, is

$$|v_{\text{ext}}| < \frac{2a}{\tau_v} \sqrt{\ln \frac{m\tau_v}{\tau}} = v_{\text{int}}. \tag{S38}$$

Similarly, when $|v_{\text{ext}}| > v_{\text{int}}$, $S v_{\text{ext}} < 0$ holds. This result is confirmed by simulation as shown in Fig.1C.

In practice, when the speed of the external input is not too large, satisfying $|v_{ext}| \ll a/\tau_v$, and that $a/(2\sqrt{2}a) \ll 1$ (which is true in practice), Eq.(S37) can be further simplified to be

$$S \approx \frac{A_u v_{ext} \tau_v}{A_{amp}} (m - \frac{\tau}{\tau_v}). \tag{S39}$$

Thus, when $m > \tau/\tau_v$, the value of S increases linearly with the external input v_{ext}, and the leading time is approximately a constant, i.e., $t_{ant} = S/v_{ext} \approx A_u \tau_v (m - \tau/\tau_v)/A_{amp}$.

REFERENCES

Fung, C., Wong, K., and Wu, S. (2010). A moving bump in a continuous manifold: A comprehensive study of the tracking dynamics of continuous attractor neural networks. *Neural Computation*, 22(3):752–792.

Mi, Y., Li, L., Wang, D., and Wu, S. (2014). A synaptical story of persistent activity with graded lifetime in a neural system. In *Advances in Neural Information Processing Systems*, pages 352–360.

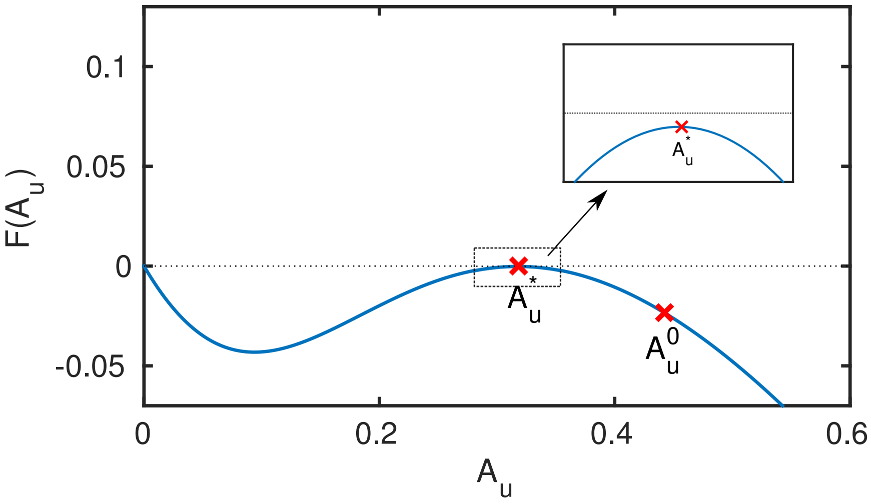

Figure S1. The mechanism for plateau decay. Parameters are chosen to be the same as the point N in Fig.1B. At A_u^*, $F(A_u^*) \approx 0$. Inset displays the fine structure around the point A_u^*. Starting from an initial state $A_u^0 > A_u^*$, it will take a considerable amount of time to cross the point A_u^*.
